# Advancing Psychiatric Safety With the Predictive Risk Identification for Mental Health Events Tool: Retrospective Cohort Study

**DOI:** 10.2196/84318

**Published:** 2026-02-06

**Authors:** Elham Dolatabadi, Valentina Tamayo Velasquez, Abdul Hamid Dabboussi, David Wen, Jennifer Crawford, Andrea E Waddell, Christo El Morr

**Affiliations:** 1 School of Health Policy and Management Faculty of Health York University Toronto, ON Canada; 2 Vector Institute Toronto, ON Canada; 3 Institute of Medical Science Temerty Faculty Medicine University of Toronto Toronto, ON Canada; 4 Waypoint Centre for Mental Health Care Penetanguishene, ON Canada; 5 Faculty of Health Sciences The University of Ontario Institute of Technology Oshawa, ON Canada; 6 Temerty Faculty of Medicine The University of Toronto Toronto, ON Canada; 7 North York General Hospital Toronto, ON Canada

**Keywords:** early warning system, psychiatric adverse events, adverse event prediction, machine learning, clinical decision support, patient safety

## Abstract

**Background:**

Patient safety incidents are a leading cause of harm in psychiatric settings, yet early warning systems (EWS) tailored to mental health remain underdeveloped. Traditional risk tools such as the Dynamic Appraisal of Situational Aggression–Inpatient Version (DASA-IV) offer limited predictive accuracy and are reactive rather than proactive.

**Objective:**

We introduce the Predictive Risk Identification for Mental Health Events (PRIME) tool, a deep learning–based EWS trained on longitudinal psychiatric electronic medical record (EMR) data to anticipate adverse events in 24-hour windows.

**Methods:**

A retrospective cohort study using routinely collected EMR data to train and validate machine learning (ML) models for short-term risk prediction was conducted. This study took place at Waypoint Centre for Mental Health Care, a large inpatient psychiatric hospital in Ontario, Canada, serving both high-security forensic and nonforensic patient populations. A total of 4651 patients and 403,098 encounters from January 2020 to August 2024 were included. For model evaluation, the 2024 test set included 900 patients and 48,313 encounters. PRIME was trained using recurrent neural networks with attention mechanisms on multivariate time-series data. The model used an autoregressive design to forecast risk based on 7 days of prior patient data and was benchmarked against the DASA-IV clinical tool and other ML baselines. The primary outcome was the occurrence of an adverse mental health event recorded in the EMR within the following 24 hours. Model performance was assessed using area under the receiver operating characteristic curve (AUC) and recall, alongside subgroup analyses and interpretability assessments using integrated gradients.

**Results:**

The long short-term memory with attention mechanism achieved the highest predictive performance (AUC=0.83), outperforming existing tools such as DASA-IV by 0.20 AUC (0.81 vs 0.61) and demonstrating the potential of ML-based models to support proactive risk management in mental health settings.

**Conclusions:**

The PRIME tool is one of the first developed and evaluated deep learning–based EWS for psychiatric inpatient care. By outperforming existing clinical tools and providing interpretable, rolling predictions, PRIME offers a pathway toward safer, more proactive mental health interventions. Future work should assess its equity implications and integration into routine psychiatric workflows.

## Introduction

Patient and staff safety are top priorities in health care, yet patient safety incidents remain the third leading cause of death in Canada [1]. Many of these incidents stem from adverse events such as falls, medication errors, and medical complications [2]. A recent study found that 1 in 4 hospital admissions involved adverse events, with a quarter of these deemed preventable [3]. While all health care settings face safety risks, psychiatric environments present a distinct set of challenges, including suicide, restraint, and seclusion—events that contribute to continued deterioration and injury [4,5]. Despite a higher prevalence of adverse events in mental health, research on patient safety and mental deterioration–related adverse events in these settings remains limited compared to other medical fields [6,7].

These incidents not only worsen patient outcomes but also increase risks for staff [8]. Worldwide, approximately 24% of health care workers experience physical violence annually, with psychiatric staff at particularly high risk [9-11]. Reducing adverse events through assessment and prediction is crucial for improving staff and patient safety.

Current methods for assessing patient deterioration rely heavily on voluntary reporting, critical incident reviews, and clinician judgment [12]. While actuarial tools such as the Dynamic Appraisal of Situational Aggression and the Brøset Violence Checklist are also used, these 2 primarily target short-term aggression and violence prediction, and they have shown limited predictive accuracy and tend to miss early warning signs [13,14]. As a result, many opportunities for timely intervention are lost, especially in high-risk but low-observable cases with early signs of deterioration that are not easily detected. Additionally, there are other widely validated measures for more specific feature prediction, such as the Historical, Clinical, and Risk Management, also used for violence risk assessment; the Columbia Suicide Severity Rating Scale for the assessment of suicidal ideation and behavior; and many other risk assessment tools [15,16]. We focused on both the Dynamic Appraisal of Situational Aggression and Brøset Violence Checklist measures as they are 2 of the most widely validated and routinely implemented structured risk assessment tools in inpatient psychiatry [17].

Early warning systems (EWSs) are widely used in medicine, leveraging routinely collected clinical data to detect early signs of patient deterioration. Tools such as the National Early Warning Score 2 have been effectively implemented in acute care settings to support timely interventions [18-20]. At the same time, machine learning (ML) is transforming risk assessment by enabling the analysis of large-scale, high-dimensional health care data [21-23]. Predictive ML models are developed using historical patient records combined with expert input to train, test, and refine algorithms for higher performance and clinical relevance [24,25]. Compelling examples include CHARTWatch, developed to predict inpatient deterioration in general internal medicine, and Sepsis Watch, designed to identify patients at risk of sepsis before clinical recognition [26-28]. However, psychiatric care has not seen comparable innovation, in part due to the complexity of mental health data, lack of validated digital tools, and underrepresentation of psychiatric settings in EWS research.

To address this gap, we introduce a novel ML-based EWS, the Predictive Risk Identification for Mental Health Events (PRIME) tool. The PRIME tool is a deep learning–based EWS leveraging longitudinal electronic medical record (EMR) data from a specialized psychiatric hospital. The goal of the PRIME tool is to predict mental health–specific adverse events, including but not limited to self-harm, suicide attempts, violence toward others, and aggressive behaviors (Multimedia Appendix 1). PRIME is trained to predict the likelihood of these adverse events within 24-hour windows using autoregressive recurrent neural networks enriched with attention mechanisms and interpretability via integrated gradients. Unlike traditional tools, PRIME is capable of continuous, real-time risk forecasting even in the absence of prior incidents. We benchmarked PRIME against Dynamic Appraisal of Situational Aggression–Inpatient Version (DASA-IV) and other ML models, and it demonstrated superior performance, particularly in complex and high-risk subgroups.

Through this study, we aimed to move beyond reactive safety practices toward proactive, data-informed risk mitigation in mental health care, advancing both patient and staff safety in a setting long underserved by digital innovation.

## Methods

### Study Design and Data Acquisition

In this study, we used routinely collected clinical data extracted from the EMR at Waypoint Centre for Mental Health Care (hereafter referred to as “Waypoint”), Ontario, Canada. We retrospectively retrieved data from all patients at Waypoint between January 2020 and August 2024, including static and dynamic variables (Multimedia Appendix 1).

### Ethical Considerations

This study was approved by the York University Office of Research Ethics (certificate e2023-163) and the Research Ethics Board of Waypoint Centre for Mental Health Care (reference #RCRA#23.08.01) with waived informed consent. The Research Ethics Board waived the need for informed consent since the data was retrospectively collected in routine practice.

### Data Representation and Processing

First, we conducted a literature review to identify factors widely associated with mental health deterioration and adverse events. We collaborated with clinicians, physicians, clinical informatics specialists, and the research team to review these factors and select the variables within our EMR (Multimedia Appendix 1). The baseline data preprocessing included one-hot encoding and normalization of all measures. We implemented a standardized aggregation strategy to address the variability in time-series data arising from differing measurement frequencies, where some clinical parameters were recorded daily and others were recorded multiple times per day. These factors encompassed a range of clinical and behavioral variables in the following categories: inpatient admission assessments that included demographic and diagnosis data, clinical risk assessments, physiological data, recent behavioral data, and mental status assessment data (Multimedia Appendix 1). Patient encounters were segmented into 24-hour intervals, aligning with clinical workflows that typically operate on daily cycles for alerts. Within each interval, all measures were aggregated to provide a comprehensive snapshot of patient health over the specified time frame. Numerical variables were averaged across the interval, whereas categorical variables were first encoded numerically based on severity or clinical importance and then summed within the 24-hour period.

We collected admission diagnosis data based on the *Diagnostic and Statistical Manual of Mental Disorders, Fifth Edition*, selecting the 45 most frequent diagnoses across all patients to prevent overfitting. Medication data included our patient group’s 5 most relevant categories, each represented as a binary indicator denoting whether it was administered within the previous 24 hours (Multimedia Appendix 1).

The primary outcome in our study was the occurrence of any mental health adverse event. For our prediction task, each patient encounter was labeled based on whether a logged adverse event in the EMR system occurred within the following 24-hour bin. This binary label (event vs no event) was used as the target variable for PRIME training. Moreover, prior adverse events in the previous 24-hour intervals were also dynamically added to future intervals, referred to as the history of any incident.

### Building the PRIME Model

To improve psychiatric-medical baselines, we designed a deep learning–based EWS. Specifically, we developed recurrent neural networks using the long short-term memory (LSTM) model that triggered an alert every 24 hours based on a variable sequence length (3-7 days) of patient data, treated as a hyperparameter. During training, the ground truth history of each adverse event was provided for every 24-hour interval in the model. In the inference phase, the model operated in an autoregressive mode: it used its own predicted output for the previous 24-hour window as an input signal for the next prediction step. To enhance the model’s ability to focus on the most relevant temporal signals within the input sequence, we further explored an LSTM model with attention mechanisms (LSTM+attention). In this variant, an attention layer was added to the LSTM hidden states Multimedia Appendix 2. For each 24-hour prediction interval, the attention mechanism dynamically assigned weights to each time step in the historical input sequence, allowing the model to selectively focus on the most informative data that contributed to the risk signal.

We also evaluated our model against 2 ML approaches: light gradient boosting machine (LightGBM) and feedforward neural network (FNN). All time-series features were aggregated over 3 to 7 days using the same methodology applied to 24-hour intervals. Predictions were then made for the next 24-hour interval, allowing for consistent model evaluation and direct comparison across different sequence lengths to identify the best-performing approach. To support robust evaluation and model selection, the dataset was first partitioned into two distinct test sets: (1) a held-out patient test set with no patient overlap between the development (3000 patients) and the test sets (751 patients) and (2) an out-of-time test set split across time using 2020 to 2022 data for training and 2023 data for testing.

Once the model selection was finalized, we performed a final training phase using all data from 2020 to 2023 to build PRIME. This final model was then evaluated on 2024 data to assess real-world applicability. Model calibration was assessed on the 2024 evaluation cohort using reliability (calibration) curves and the Brier score [29,30]. Reliability curves were generated using 10 uniformly spaced probability bins plotting the mean predicted risk against observed outcome frequency within each bin. The Brier score was computed as the mean squared difference between predicted probabilities and observed binary outcomes, providing a quantitative measure of overall probabilistic accuracy.

To explore the factors driving PRIME’s predictions, we used integrated gradients to compute feature importance in our LSTM model by computing the path-integrated gradients from the input to the actual output [31]. To quantify uncertainty, gradients were bootstrapped over 100 resampled datasets.

### Comparison With Clinical Measures

We compared PRIME’s predictive performance against that of the DASA-IV, a standardized tool used at our hospital to evaluate risks of aggression [32]. The PRIME tool’s predictions included all mental health–specific adverse events recorded in the hospital’s incident log (Multimedia Appendix 1). DASA-IV includes 7 items assessing behavioral indicators (ie, irritability, negative attitudes, and verbal threats), each scored as 0 (not observed) or 1 (observed), with a total score categorized as low (0-1), moderate (2-3), or high (>3) [33,34]. To align DASA-IV with PRIME’s binary classification, we restructured the risk categories. Moderate and high risk were grouped as “at risk” (positive prediction), whereas low risk was grouped as “no risk” (negative prediction). PRIME is designed to predict a broader range of mental health–specific adverse events, whereas DASA-IV is limited to aggression-related incidents and deterioration. Our goal was to compare the PRIME tool with the current validated tool used in clinical practice. This allowed us to compare DASA-IV’s performance against PRIME’s predictions and the ground truth outcomes recorded in patient encounters.

## Results

### Cohort Characteristics

The dataset encompassed 4651 patients and 403,098 patient encounters over 55 months. The demographic characteristic distribution of the patient cohort is presented in Table 1. For the evaluation of the best-performing ML model and comparison against clinical baselines, we used data from 2024, with detailed breakdowns provided in Table 1.

**Table 1:** Cohort characteristics and dataset splits used for model development and evaluation. After final model selection, the full dataset from 2020 to 2023 (model development) was used to train the Predictive Risk Identification for Mental Health Events, which was evaluated using 2024 (model evaluation) data to assess real-world performance.

			Model development (2020-2023)									Model evaluation (2024)—evaluation set (48,313)
			Held-out patients				Out-of-time patients					
Data split (number of patient encounters)			Development set (281,022)		Test set (73,763)		Development set (259,257)		Test set (95,528)			
Patients, n (%)			3000 (80)		751 (20)		2851 (70.3)		1202 (29.7)		900 (100)	
Period			January 1, 2020, to December 31, 2023		January 1, 2020, to December 31, 2023		January 1, 2020, to December 31, 2022		January 1, 2023, to December 31, 2023		January 1, 2024, to August 19, 2024	
LOS^a^, mean (SD)			629.22 (632.67)		530.41 (654.51)		708.04 (676.56)		336.90 (390.40)		134.99 (117.83)	
**Sex, n (%)**												
	Female	839 (27.98)		231 (30.81)		845 (29.63)		310 (25.75)		235 (26.05)		
	Male	2045 (68.17)		502 (66.82)		1912 (67.08)		842 (70.05)		633 (70.37)		
	Other	116 (3.85)		18 (2.37)		94 (3.29)		50 (4.20)		32 (3.58)		
**Sexual orientation, n (%)**												
	Heterosexual	1878 (62.59)		472 (62.90)		1833 (64.31)		699 (58.15)		541 (60.14)		
	Other	1122 (37.41)		279 (37.10)		1018 (35.69)		503 (41.85)		359 (39.86)		
**Race, n (%)**												
	Black	273 (9.10)		36 (4.73)		224 (7.85)		109 (9.03)		73 (8.10)		
	First Nations	61 (2.05)		18 (2.45)		63 (2.21)		23 (1.92)		19 (2.13)		
	White	1987 (66.23)		572 (76.20)		2000 (70.15)		763 (63.49)		570 (63.28)		
	Other races	679 (22.62)		125 (16.62)		564 (19.78)		307 (25.57)		238 (26.49)		
**Incident prevalence**												
	Total number of incidents	11,744		2569		10,688		3625		2106		
	Patients, n (%)	762		209		766		342		266		

^a^LOS: length of stay in days.

Adverse event distribution per individual varied across the sample of patients between 2020 to 2024. When grouping the number of patients by the frequency of adverse events they experienced during their hospital stay, there is a decrease in the number of patients who experience a high count of adverse events. Most patients experienced few or no adverse events: 69.9% (3251/4651) had no incidents, and 12.6% (587/4651) experienced up to 2 incidents. A total of 7.4% (344/4651) of the patients had between 3 and 16 events, with a median of 14.5 (IQR 14.25). A smaller group of 162 patients experienced between 17 and 83 incidents, most of whom (n=42, 25.9%) had between 17 and 20, whereas only 22 (13.6%) had more than 83 events. As incident frequency increased, cohort size decreased. The mean number of adverse events across the sample was 2.85, whereas the mode and median were both 0, highlighting the skewed nature of the data. This imbalance is important to consider as it affects how the model learns from the hospital’s patient population, with most of the training data representing patients with few or no incidents.

### PRIME’s Predictions

Table 2 presents the performance comparison of the 4 ML models: light gradient boosting (LightGBM), feedforward neural network (FNN), LSTM, and LSTM+attention. Each model was trained multiple times using different random seeds, and performance metrics were averaged across runs to ensure robustness. Given the imbalanced nature of the dataset, model performance was evaluated using the area under the receiver operating characteristic curve (AUC) and recall. The LSTM+attention model consistently achieved the highest performance, with an AUC of 0.87 for held-out patients and 0.72 for out-of-time patients Multimedia Appendix 3. We selected the LSTM+attention model as the final architecture for PRIME (Table 2).

**Table 2:** Performance comparison of 4 machine learning models evaluated using area under the receiver operating characteristic curve (AUC) and recall. Metrics were averaged across multiple runs with different random seeds to ensure robustness.

Category and subcategory			AUC	Recall
**Model selection, mean (SD)**				
	**Held-out patients**			
		LightGBM^a^	0.51 (0.004)	0.02 (0.009)
		FNN^b^	0.52 (0.005)	0.05 (0.011)
		LSTM^c^	0.87 (0.002)	0.75 (0.02)
		LSTM+attention	0.87 (0.002)	0.74 (0.04)
	**Out-of-time patients**			
		LightGBM	0.52 (0.002)	0.04 (0.003)
		FNN	0.54 (0.01)	0.08 (0.03)
		LSTM	0.84 (0.01)	0.72 (0.01)
		LSTM+attention	0.85 (0.01)	0.75 (0.02)
**PRIME’s^d^ performance**				
	**Sex**			
		Male	0.83	0.36
		Female	0.84	0.29
		Intersex	0.87	0.23
	**Race**			
		Black	*0.69* ^e^	0.16
		First Nations	0.8	0.16
		White	0.84	0.38
		Other racial identities	*0.81*	0.27
	**Sexual orientation**			
		Heterosexual	0.82	0.34
		Other	0.84	0.34
	**Program type**			
		Regional (nonforensic)	0.83	0.34
		Provincial (forensic)	0.8	0.27
	**Age group (years)**			
		18-65	0.81	0.32
		≥65	0.81	0.38
	All		0.81	0.3

^a^LightGBM: light gradient boosting machine.

^b^FNN: feedforward neural network.

^c^LSTM: long short-term memory.

^d^PRIME: Predictive Risk Identification for Mental Health Events.

^e^Italicization indicates significance.

For the evaluation using the dataset from 2024, with 48,313 encounters and 2106 recorded adverse events, PRIME achieved an AUC of 83% (Table 2). The performance varied within and across subgroups, with AUC ranging from 0.69 (Black patients) to 0.87 (intersex patients), indicating potential biases favoring larger, more represented groups within the sample data. Across racial subgroups, AUC differed by 14%; across sex subgroups, AUC varied by 5%; across sexual orientation subgroups, AUC varied by 2%; across program types, AUC differed by 4%; and, across age groups, AUC variation was minimal (<1%).

Calibration analysis demonstrated that PRIME produced well-aligned risk estimates. The reliability curve closely followed the identity line across predicted probability bins (Multimedia Appendix 4), indicating good agreement between predicted and observed event rates. The model achieved a Brier score of 0.036 on the evaluation set, reflecting strong overall calibration performance given the low event prevalence.

**Figure 1:**
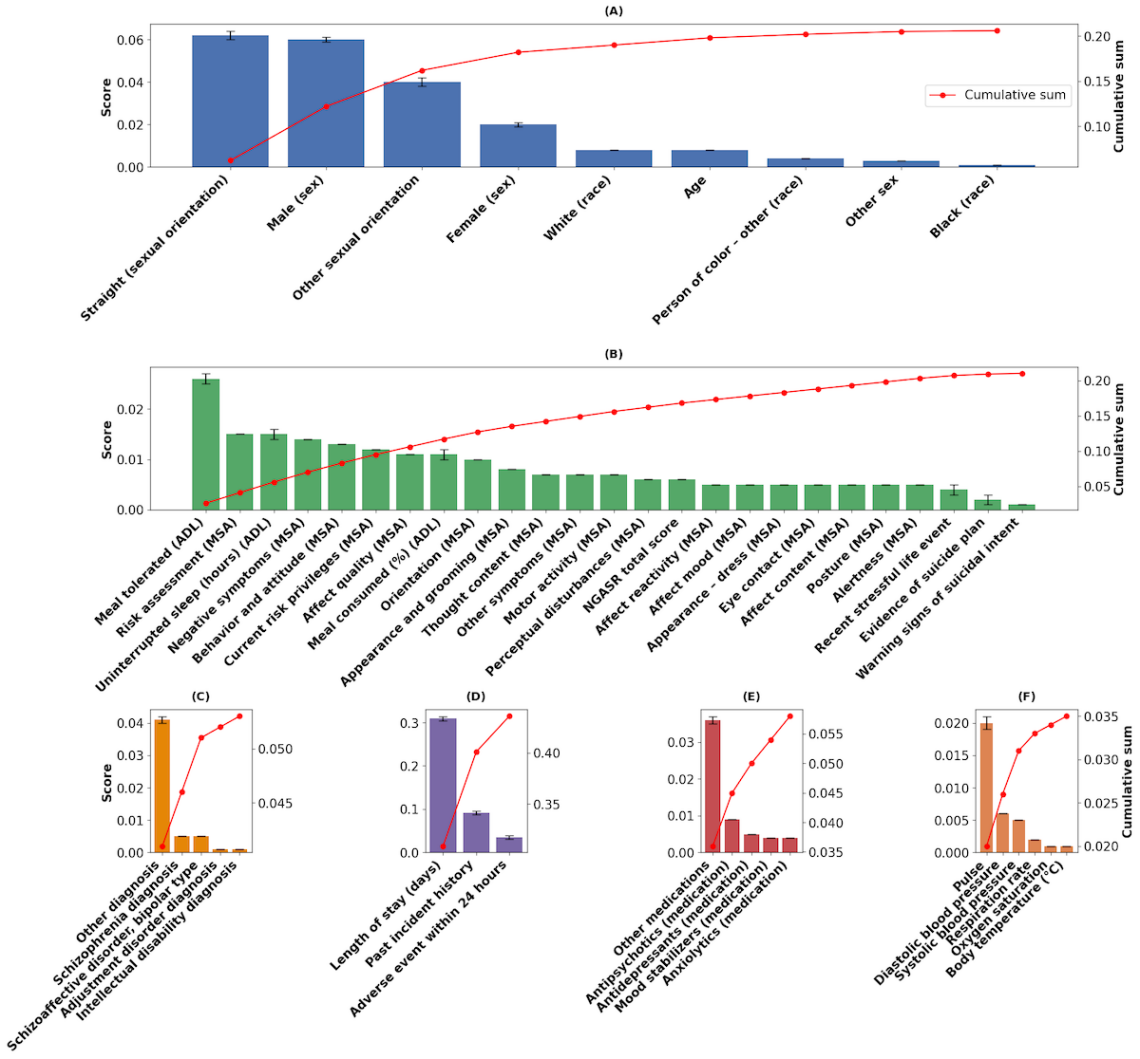
Feature importance across distinct categories in the PRIME model: (A) Demographic features including gender, race, and sexual orientation; (B) Clinical assessment features such as mental status indicators and functional assessments; (C) Clinical diagnoses including major psychiatric conditions, including schizophrenia diagnosis; (D) Clinical variables related to adverse events, incident history, and hospital stay duration; (E) Medication-related features, including mood stabilizers and antipsychotics; and (F) Vital signs, including pulse, blood pressure, and oxygen saturation.

Feature importance was aggregated across time steps and encounters and summarized at the feature level (Figure 1). Integrated gradient attributions were bootstrapped over 100 resampled evaluation datasets, with the resulting variability visualized as error bars. Ranking stability was assessed using Spearman correlation, demonstrating near-perfect robustness (ρ=0.99, –0.0001 to +0.0001). Among the 40 features included in PRIME, the top 16 predictors accounted for approximately 80% of the model’s total importance, reflecting a diverse combination of demographic, medical, and psychosocial factors that drive risk prediction.

When further analyzing feature contributions within specific categories, demographic factors (Figure 1A) and indicators, with heterosexual sexual orientation and male sex showing the largest individual contributions, followed by *other* sexual orientation categories and female sex. Race-related variables and age demonstrated comparatively smaller effects. From the clinical assessments (Figure 1B), meal tolerated (ADL), risk assessment (MSA) and uninterrupted sleep (ADL) were identified as important contributors. In the category of clinical diagnoses (Figure 1C), schizophrenia and schizoaffective or bipolar disorder emerged as the most significant predictors. Among clinical variables (Figure 1D), length of hospital stay emerged as the most influential contributor, followed by history of past incidents and adverse events in the previous 24 hours. In the medication category (Figure 1E), the other medications category exhibited the largest overall contribution, followed by antipsychotics and antidepressants, with mood stabilizers and anxiolytics contributing more modestly. Finally, among vital signs (Figure 1F), features such as pulse, blood pressure, and oxygen saturation met the 0.90 cumulative importance threshold, although their influence remained relatively modest.

### Comparison With the Standardized Risk Assessment Tool DASA-IV

**Figure 2:**
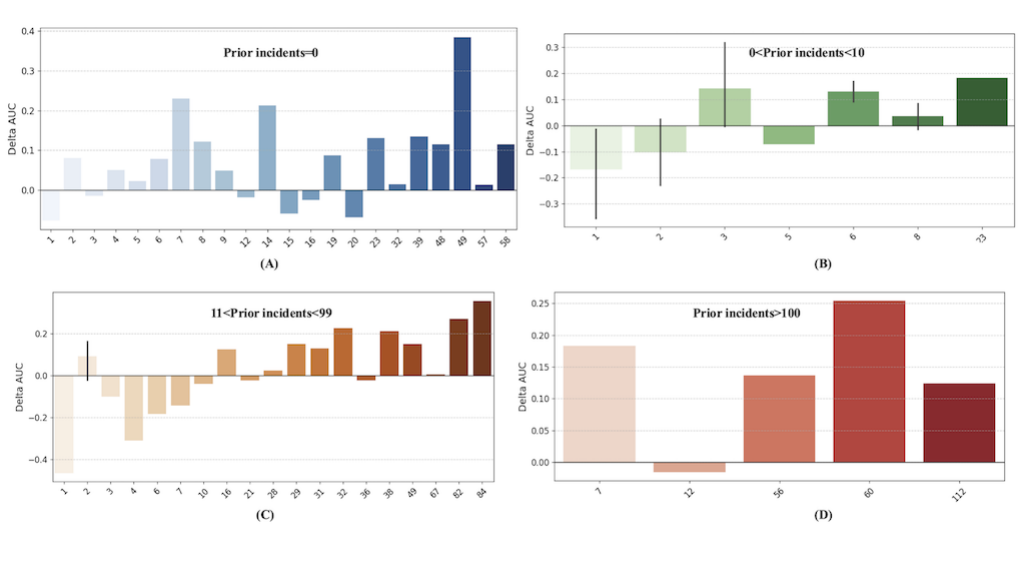
Difference in AUC ROC performance scores (Delta AUC = ML AUC ROC - DASA AUC ROC). A positive delta AUC indicates the ML model outperformed DASA for that specific cohort group. A negative delta AUC indicates DASA outperformed ML for that specific cohort group. AUC: area under the receiver operating characteristic curve; DASA: Dynamic Appraisal of Situational Aggression; ML: machine learning; ROC: receiver operating characteristic curve.

PRIME demonstrated a 0.2 AUC improvement over the DASA-IV assessment tool when assessed on the 2024 evaluation dataset, with PRIME achieving an AUC of 0.81 compared to DASA-IV’s AUC of 0.61 (Multimedia Appendix 5). To further assess PRIME’s performance across different patient groups, we analyzed its effectiveness based on the historical incidence of adverse events for each individual in the training dataset (previous adverse event history). Figure 2 illustrates the performance differences between PRIME and DASA-IV across various patient groups, where each group is defined by the number of adverse events recorded in both the training (past) and evaluation (future) datasets. To examine the model’s performance compared to that of DASA-IV, we defined subgroups based on all the unique combinations of adverse event occurrences observed in the training and test datasets. This yielded 63 unique subgroups representing different patterns and combinations of past and future incident frequencies across the datasets.

The PRIME tool significantly outperformed DASA-IV in 40 of the 63 subgroups (Wilcoxon test; *P*=.007). For individuals with no prior incidents in the training set but up to 58 total incidents in the evaluation period (Figure 2A), PRIME achieved an AUC of 0.62, whereas DASA-IV achieved an AUC of 0.50. For individuals with up to 10 incidents in the past and up to 23 in the future (Figure 2B), DASA-IV outperformed PRIME in cases in which individuals had 1, 2, or 5 future incidents. However, PRIME outperformed DASA-IV in the remaining 4 subcategories within this range. For individuals with moderate incident frequency (11-99 past incidents), PRIME outperformed DASA-IV in 11 of the 19 groups (Figure 2C). Among individuals with frequent incidents (>100 past incidents), PRIME outperformed DASA-IV in 4 of the 5 subgroups (Figure 2D). Notably, PRIME’s performance was better in edge cases in which individuals had a high number of past incidents but only 1 in the future.

## Discussion

Despite the growing number of adverse events in mental health settings, deep learning tools that leverage routinely collected EMR data to predict patient deterioration remain limited. Our model, PRIME, represents a first-of-its-kind approach tailored specifically to psychiatry and demonstrated strong predictive performance, achieving an AUC of 0.83. Leveraging autoregressive LSTM with attention mechanisms, PRIME operates in a rolling prediction mode, enabling 24-hour forecasts even in the absence of recent incident data. Notably, the history of prior incidents emerged as one of the most informative features, reinforcing the predictive value of temporal continuity in patient risk trajectories. Furthermore, the inclusion of patients from both forensic and nonforensic acute care programs contributes to the model’s generalizability across diverse mental health populations. The strong calibration performance observed for PRIME is particularly important for clinical deployment, where accurate probability estimates are essential for risk stratification and decision support. Well-calibrated predictions enable clinicians to interpret PRIME scores as meaningful risk estimates rather than solely as ranking signals.

Currently, no ML-based predictive alerting tools are deployed in mental health settings. Instead, clinicians rely on actuarial tools such as DASA-IV to assess risks related to violence and aggression [35]. On the same dataset, PRIME outperformed DASA-IV (AUC=0.83 vs 0.61). While DASA-IV has reported AUCs between 0.61 and 0.82 in other studies, it is important to note that PRIME and DASA-IV target different outcomes [36]. PRIME captures a broader spectrum of deterioration events, including suicide, self-harm, and clinical decompensation, whereas DASA-IV is limited to aggression-related outcomes. The lower AUC for DASA-IV in our dataset likely reflects these differences in scope. Nonetheless, PRIME’s ability to deliver significantly stronger performance across a wider range of adverse events underscores its versatility and robustness. In clinical practice, focusing solely on aggression is insufficient; risks of suicide and self-harm are equally critical. By encompassing a more comprehensive set of risks, PRIME provides clinicians with a holistic and actionable risk assessment framework, supporting earlier and more effective interventions. PRIME also showed strong performance even in patients with no prior recorded incidents, addressing a critical limitation of traditional tools that rely heavily on observable behavior or clinician judgment.

The feature “adverse event in the past 24 hours” emerged as one of the predictors of future deterioration, consistent with findings from acute care settings where recent clinical instability is a key driver of risk. Similar patterns have been observed in inpatient deterioration models, where temporal proximity to prior events significantly enhances predictive accuracy [37,38]. Beyond clinical history, our results indicate that a wide array of features, including demographic variables, mental status assessments, clinical diagnoses, medications, and vital signs, contribute meaningfully to risk prediction. This multidimensional pattern aligns with emerging work suggesting that accurate prediction of psychiatric outcomes requires integrating different types of structured medical data and psychosocial factors [39-41]. Overall, these findings underscore the importance of using holistic patient representations to capture the complex drivers of risk in mental health, a direction that has been underexplored in existing ML applications in psychiatry.

A limitation of this study, as previously noted, is the underrepresentation of certain demographic subgroups, which affected the model’s predictive performance. We observed up to an 18% variation in AUC across subpopulations, indicating disparities in performance. Notably, the model was less accurate for 2 racial subgroups: Black and First Nations individuals, with AUC scores 14% and 3% lower, respectively, than those for the overall model performance. Additionally, both groups had a recall of 0.16, which was lower than that of all other subgroups, suggesting a higher rate of false negatives and an undercalling of risk. These disparities likely stem from the low representation of these groups in the dataset, with Black individuals comprising less than 10% (309/3751) of the sample in the training set and less than 10% (73/900) in the evaluation set. Similarly, First Nations individuals comprise less than 3% (79/3751) in the training set and less than 3% (19/900) in the evaluation set. Furthermore, this study did not assess the intersectional effects, such as whether the demographic factors had any effect or potential differences between the forensic and nonforensic programs. These represent important assessments for future work to further evaluate whether predictive models such as PRIME are unbiased and generalizable across different clinical settings and patient populations.

Additionally, while the PRIME tool demonstrated high predictive performance, the complexity that is inherently present in deep learning models may limit clinical interpretability. Ensuring clinician confidence and understanding of the model’s prediction is critical for successful implementation. Ongoing monitoring and evaluation of PRIME are needed to assess its real-world performance and potential biases.

Future work will evaluate the utility, feasibility, and efficacy of the PRIME tool in real-world clinical settings. This future work will also focus on mitigating the previously mentioned biases through bias-aware data augmentation and fairness-aware learning algorithms (eg, adversarial debiasing) to improve representation across subgroups [42-44]. Piloting the PRIME tool in a live clinical setting is the next step in validating its performance and efficacy and informing the next steps toward broader clinical deployment. In our future pilot and deployment, we plan to use PRIME as a binary risk assessment tool to flag patients at a high risk of adverse events in mental health settings. Finally, although the PRIME tool was developed using data from a single mental health hospital, the model framework and variable-mapping methodological approaches are transferable to other mental health and psychiatric settings. If the PRIME tool is to be implemented in other settings, it will require retraining and validation to account for different patient populations, data sources, and documentation practices.

In this study, we developed and evaluated an LSTM model that could predict patients at risk of an adverse event. The model showed good performance across different subgroup populations, and our findings suggest that the model would outperform currently used risk assessment tools. Its autoregressive design, model evaluation, and near–real-time operation position it for real-world clinical integration. By generating dynamic forecasts without dependence on manual clinician input, PRIME can augment existing workflows and support earlier interventions in settings where mental health staff face high demands and elevated safety risks.

## Appendix

Multimedia Appendix 1 List of baseline and temporal features used for the PRIME (Predictive Risk Identification for Mental Health Events) tool.

Multimedia Appendix 2 Hyperparameters for the long short-term memory model and the long short-term memory+attention model. A comparison of the hyperparameter configurations for the two top performing models evaluated.

Multimedia Appendix 3 A summary of the model’s performance metrics across 18 epochs. Early stopping triggered at epoch 19.

Multimedia Appendix 4 Calibration (reliability) curve for the PRIME model evaluated on the 2024 test dataset.

Multimedia Appendix 5 Clinical baseline model: Dynamic Appraisal of Situational Aggression’s Predictive Performances across different data splits (area under the receiver operating characteristic curve).

## Abbreviations

AUC: area under the receiver operating characteristic curve
DASA-IV: Dynamic Appraisal of Situational Aggression–Inpatient Version
EMR: electronic medical record
EWS: early warning system
LSTM: long short-term memory
ML: machine learning
PRIME: Predictive Risk Identification for Mental Health Events

## Data Availability

The datasets generated or analyzed during this study are not publicly available due to privacy policies and ethical restrictions but are available from the corresponding author on reasonable request.
