# Supplementary material for: Advancing Psychiatric Safety With the Predictive Risk Identification for Mental Health Events Tool: Retrospective Cohort Study
Source: JMIR Ment Health. 2026 Feb 6;13:e84318. doi: 10.2196/84318 (PMC12924039; doi:10.2196/84318)
Supplement: Multimedia Appendix 1 [file mental_v13i1e84318_app1.docx]

**Multimedia Appendix 1**

### S-Table 1**. List of baseline and temporal features used for the PRIME tool**

| **Included Variables** | **Variable (Location of data element in EMR)** | **Static/Dynamic Measure** | **Type of Variable** |  |
| --- | --- | --- | --- | --- |
| **Demographics** | | | |  |
| **Inpatient Admission Assessment** | Sexual Orientation: Straight, Other sexual orientations | Static | Numerical |  |
|  |  |  |  |  |
|  | Sex: Male, Female, Intersex |  | Numerical |  |
|  |  |  |  |  |
|  |  |  |  |  |
|  | Race: White, Black, People of colour, Indigenous |  | Numerical |  |
|  |  |  |  |  |
|  |  |  |  |  |
|  |  |  |  |  |
|  | Age |  | Discrete (Numerical) |  |
| **Clinical** | | | |  |
| **Medication (Administration)** | Generic mood stabilizers | Dynamic | Binary (0 or 1) |  |
|  | Generic antipsychotics |  |  |  |
|  | Generic antidepressants |  |  |  |
|  | Generic anxiolytics |  |  |  |
|  | Other |  |  |  |
| **Vital Signs** | Systolic blood pressure | Dynamic | Continuous (Numerical) |  |
|  | Diastolic blood pressure |  |  |  |
|  | Pulse |  | Continuous (Numerical) |  |
|  | Temperature in Celsius |  | Continuous (Numerical) |  |
|  | Respiration rate |  | Continuous (Numerical) |  |
|  | Oxygen saturation level |  | Continuous (Numerical) |  |
| **Length of stay** | Length of stay (days) | Dynamic (input in model development from dataset was static) | Discrete (Numerical) |  |
| **Primary Diagnosis (Top 45)** | Schizophrenia | Dynamic | Categorical |  |
|  | Autism spectrum disorder |  |  |  |
|  | Intellectual developmental disorde (intellectual disability), Severe |  |  |  |
|  | Schizoaffective disorder, Bipolar type |  |  |  |
|  | Intellectual developmental disorder (intellectual disability), Moderate |  |  |  |
|  | Adjustment disorders, with mixed disturbance of emotions and conduct |  |  |  |
|  | Intellectual developmental disorder (intellectual disability), Mild |  |  |  |
|  | Major neurocognitive disorder due to multiple etiologies, Moderate, with agitation (code first the other medical etiologies) |  |  |  |
|  | Major neurocognitive disorder due to multiple etiologies, Severe, with agitation (code first the other medical etiologies) |  |  |  |
|  | Bipolar I disorder, Current or most recent episode depressed, in full remission |  |  |  |
|  | Major neurocognitive disorder due to multiple etiologies, with behavioral disturbance |  |  |  |
|  | Bipolar I disorder, Current or most recent episode unspecified |  |  |  |
|  | Bipolar I disorder, Current or most recent episode manic, Moderate |  |  |  |
|  | Unspecified schizophrenia spectrum and other psychotic disorder |  |  |  |
|  | Schizoaffective disorder, Depressive type |  |  |  |
|  | Major neurocognitive disorder due to Huntington's disease, with behavioral disturbance (code first G10 Huntington's disease) |  |  |  |
|  | Bipolar I disorder, Current or most recent episode depressed, in partial remission |  |  |  |
|  | Alcohol use disorder, Severe |  |  |  |
|  | Bipolar I disorder, Current or most recent episode manic, with psychotic features |  |  |  |
|  | Bipolar I disorder, Current or most recent episode depressed, Moderate |  |  |  |
|  | Major neurocognitive disorder probably due to vascular disease, Moderate, with agitation (no additional medical code) |  |  |  |
|  | Borderline personality disorder |  |  |  |
|  | Other specified schizophrenia spectrum and other psychotic disorder |  |  |  |
|  | Alcohol use disorder, Moderate |  |  |  |
|  | Posttraumatic stress disorder |  |  |  |
|  | Antisocial personality disorder |  |  |  |
|  | Major neurocognitive disorder due to probable frontotemporal degeneration, with behavioral disturbance (code first G31.09 frontotemporal degeneration) |  |  |  |
|  | Major neurocognitive disorder due to probable Alzheimer's disease, with behavioral disturbance (code first G30.9 Alzheimer's disease) |  |  |  |
|  | Cannabis-induced psychotic disorder, with moderate or severe use disorder |  |  |  |
|  | Major neurocognitive disorder due to another medical condition, with behavioral disturbance |  |  |  |
|  | Persistent depressive disorder |  |  |  |
|  | Major neurocognitive disorder due to possible Alzheimer's disease, Severe, with agitation (no additional medical code) |  |  |  |
|  | Major depressive disorder, Recurrent episode, Moderate |  |  |  |
|  | Major neurocognitive disorder due to prion disease, Unspecified severity, Without accompanying behavioral or psychological disturbance (code first A81.9 prion disease) |  |  |  |
|  | Major depressive disorder, Recurrent episode, with psychotic features |  |  |  |
|  | Adjustment disorders, with depressed mood |  |  |  |
|  | Major neurocognitive disorder due to multiple etiologies, Unspecified severity, Without accompanying behavioral or psychological disturbance (code first the other medical etiologies) |  |  |  |
|  | Bipolar I disorder, Current or most recent episode hypomanic, Unspecified |  |  |  |
|  | Major neurocognitive disorder possibly due to vascular disease, Moderate, with other behavioral or psychological disturbance (no additional medical code) |  |  |  |
|  | Major depressive disorder, Recurrent episode, Severe |  |  |  |
|  | Major neurocognitive disorder due to another medical condition, Moderate, with agitation (code first the other medical condition) |  |  |  |
|  | Bipolar I disorder, Current or most recent episode manic, Severe |  |  |  |
|  | Major neurocognitive disorder due to probable frontotemporal degeneration, Unspecified severity, Without accompanying behavioral or psychological disturbance (code first G31.09 frontotemporal degeneration) |  |  |  |
|  | Adjustment disorders, with mixed anxiety and depressed mood |  |  |  |
|  | Cocaine use disorder, Moderate |  |  |  |
| **Assessments** | | | |  |
| **Mental Status Assessment (MSA)** | Dress (Appearance) | Dynamic | Discrete (Numerical) |  |
|  | Eye contact (Appearance) |  | Discrete (Numerical) |  |
|  | Posture (Appearance) |  | Discrete (Numerical) |  |
|  | Grooming (Appearance) |  | Discrete (Numerical) |  |
|  | Orientation (Sensorium and cognition) |  | Discrete (Numerical) |  |
|  | Alertness (Sensorium and cognition) |  | Discrete (Numerical) |  |
|  | Attitude during interaction (Behaviour) |  | Discrete (Numerical) |  |
|  | Motor activity (Behaviour) |  | Discrete (Numerical) |  |
|  | Affect congruent to content (Affect) |  | Discrete (Numerical) |  |
|  | Affect congruent to mood (Affect) |  | Discrete (Numerical) |  |
|  | Affect quality (Affect) |  | Discrete (Numerical) |  |
|  | Affect range and intensity (Affect) |  | Discrete (Numerical) |  |
|  | Current risks (Current risks) |  | Discrete (Numerical) |  |
|  | Outcome privileges (Current risks) |  | Discrete (Numerical) |  |
|  | Negative symptoms (Symptoms checklist) |  | Discrete (Numerical) |  |
|  | Other mood disturbance indications (Symptoms checklist) |  | Discrete (Numerical) |  |
|  | Thought content (Thought content) |  | Discrete (Numerical) |  |
|  | Perceptual disturbances (Perceptual disturbance) |  | Discrete (Numerical) |  |
| **Activities of Daily Living (ADL)** | Meal percent consumed (Meal) | Dynamic | Continuous (Numerical) |  |
|  | Meal tolerance (Meal) |  | Discrete (Numerical) |  |
|  | Hours of uninterrupted sleep (Sleep) |  | Discrete (Numerical) |  |
| **Nurses' Global Assessment of Suicidal Risk (NGASR)** | Evidence of a plan to commit suicide | Dynamic (*does not change daily) | Discrete (Numerical) |  |
|  | Warning of suicidal intent |  | Discrete (Numerical) |  |
|  | Recent stressful life events – e.g., job loss, financial worries, pending court action, ect. |  | Discrete (Numerical) |  |
|  | NGASR Score |  | Continuous (Numerical) |  |
| **Adverse event log** | - Aggressive behaviour - Self harm - Elopement - Behaviour R/T Index off - Inappropriate behaviour - Breach of disposition - Violence towards others - Substance use - Contraband possession - Environmental - Weapons - Threatening behaviour - Choking - Fall - External - Adverse drug reaction - Medical device incident | | |  |
| **24-Hour-Log** | Incident log (Whether a patient has had an adverse event/ incident or not in the past 24 hours) | Dynamic | Binary (0 or 1) |  |
| **Historic Incident** | Patient’s entire LOS history of an incident (Sum of incidents log) | Dynamic | Discrete (Numerical) |  |
