# Supplementary material for: Advancing Psychiatric Safety With the Predictive Risk Identification for Mental Health Events Tool: Retrospective Cohort Study
Source: JMIR Ment Health. 2026 Feb 6;13:e84318. doi: 10.2196/84318 (PMC12924039; doi:10.2196/84318)
Supplement: Multimedia Appendix 2 [file mental_v13i1e84318_app2.docx]

**Multimedia Appendix 2**

## Model Training and Hyperparameters

We trained two variants of our model: a baseline LSTM and an LSTM enhanced with attention (LSTM+Attn). The input sequences were of length 4, with a batch size of 128 for all experiments. Both models were trained for a maximum of 100 epochs using the Adam optimizer and an initial learning rate of 0.0001. Learning rate scheduling was applied using a ReduceLROnPlateau strategy with a patience of 3 epochs and a reduction factor of 0.1. Early stopping was also used with a patience of 5 epochs, based on validation loss. The classification threshold was set at 0.5 throughout.

For the LSTM, we used a single-layer configuration with a hidden size of 4 and a dropout probability of 0.4. The LSTM+Attn model shared the same architecture but included an additional attention mechanism with 4 attention heads.

[S-Table 2](#_heading=h.745yza21g68y) summarizes the hyperparameter configurations used for each model. Model training and evaluation were conducted using a consistent data split across training, validation, and test sets.

### **S-Table 2**. Hyperparameters for the LSTM model and the LSTM+Attn model. A comparison of the hyperparameter configurations for the two top performing models evaluated.

| **Hyperparameters** | **LSTM** | **LSTM+Attn** |
| --- | --- | --- |
| Hidden Size | 4 | 4 |
| Number of Heads | N/A | 4 |
| Number of Layers | 1 | 1 |
| Drop Out Probability | 0.4 | 0.4 |
| Initial Learning Rate | 0.0001 | 0.0001 |
| ReduceLROnPlateau Patience | 3 | 3 |
| ReduceLROnPlateau Factor | 0.1 | 0.1 |
| Number of Epochs | 100 | 100 |
| Default Threshold | 0.5 | 0.5 |
| Early Stopping Patience | 5 | 5 |
