# Supplementary material for: Advancing Psychiatric Safety With the Predictive Risk Identification for Mental Health Events Tool: Retrospective Cohort Study
Source: JMIR Ment Health. 2026 Feb 6;13:e84318. doi: 10.2196/84318 (PMC12924039; doi:10.2196/84318)
Supplement: Multimedia Appendix 3 [file mental_v13i1e84318_app3.docx]

**Multimedia Appendix 3**

[Table S-3](https://docs.google.com/document/d/1YBTi0oNaor30608TaeCNbt8r4r1LAa3D/edit#heading=h.fzdy9jqhhybl) presents the performance metrics for the LSTM model over 18 epochs. Notably, the model's AUC-ROC and F1 score steadily improved until early stopping was triggered at epoch 19. The AUC-ROC reached a peak of 0.9292, with an F1 score of 0.4611, suggesting robust discriminatory ability despite the compact model architecture.

### **S-Table 3. Epoch Training Progress.** A summary of the model’s performance metrics across 18 epochs. Early stopping triggered at epoch 19.

| **Epoch Number** | **Training Loss** | **Validation Loss** | **Recall** | **AUC-ROC** | **F1 Score** |
| --- | --- | --- | --- | --- | --- |
| 1/100 | 0.4777 | 0.1586 | 0.3156 | 0.5186 | 0.0745 |
| 2/100 | 0.2034 | 0.1398 | 0.0270 | 0.6394 | 0.0332 |
| 3/100 | 0.1823 | 0.1356 | 0.0258 | 0.7262 | 0.0321 |
| 4/100 | 0.1689 | 0.1336 | 0.0071 | 0.7720 | 0.0124 |
| 5/100 | 0.1565 | 0.1322 | 0.0018 | 0.8063 | 0.0036 |
| 6/100 | 0.1453 | 0.1295 | 0.0038 | 0.8347 | 0.0076 |
| 7/100 | 0.1329 | 0.1274 | 0.0830 | 0.8649 | 0.1496 |
| 8/100 | 0.1210 | 0.1318 | 0.2269 | 0.8894 | 0.3455 |
| 9/100 | 0.1122 | 0.1286 | 0.2968 | 0.9066 | 0.4164 |
| 10/100 | 0.1067 | 0.1287 | 0.3335 | 0.9167 | 0.4474 |
| 11/100 | 0.1030 | 0.1297 | 0.3470 | 0.9235 | 0.4579 |
| 12/100 | 0.1012 | 0.1291 | 0.3516 | 0.9265 | 0.4613 |
| 13/100 | 0.1008 | 0.1291 | 0.3500 | 0.9271 | 0.4586 |
| 14/100 | 0.1005 | 0.1290 | 0.3497 | 0.9279 | 0.4596 |
| 15/100 | 0.0999 | 0.1291 | 0.3514 | 0.9290 | 0.4608 |
| 16/100 | 0.1000 | 0.1293 | 0.3514 | 0.9292 | 0.4598 |
| 17/100 | 0.1001 | 0.1291 | 0.3526 | 0.9288 | 0.4611 |
| 18/100 | 0.1001 | 0.1291 | 0.3498 | 0.9288 | 0.4586 |
