# Supplementary material for: Advancing Psychiatric Safety With the Predictive Risk Identification for Mental Health Events Tool: Retrospective Cohort Study
Source: JMIR Ment Health. 2026 Feb 6;13:e84318. doi: 10.2196/84318 (PMC12924039; doi:10.2196/84318)
Supplement: Multimedia Appendix 4 [file mental_v13i1e84318_app4.docx]

**Multimedia Appendix** **4**

**Calibration Analysis**


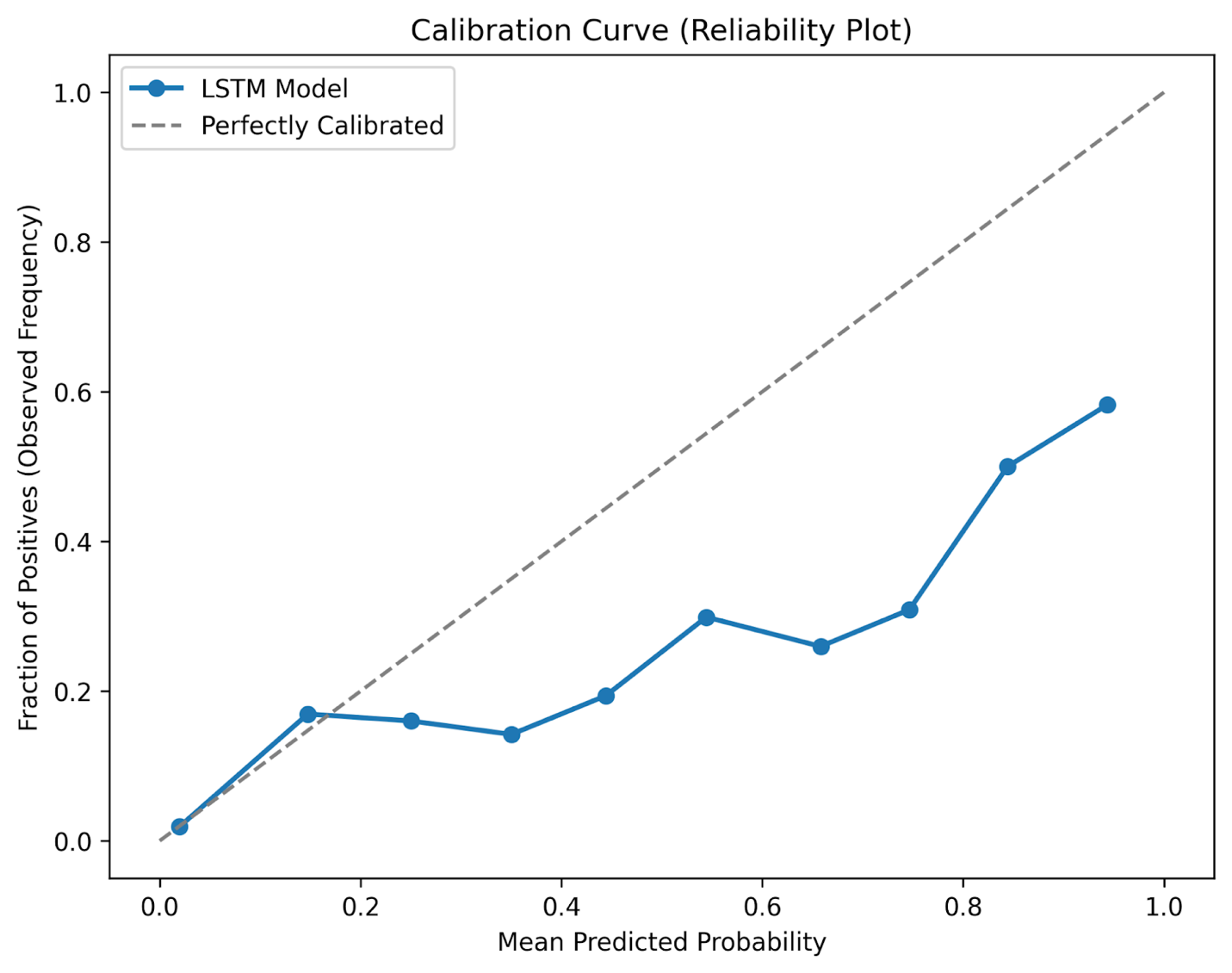


### **S-Figure 2. Calibration (reliability) curve for the PRIME model evaluated on the 2024 test dataset. Predicted probabilities were divided into 10 uniform bins. Each point represents the mean predicted risk versus the observed event frequency within a bin. The dashed diagonal line indicates perfect calibration.**
