# Supplementary material for: Advancing Psychiatric Safety With the Predictive Risk Identification for Mental Health Events Tool: Retrospective Cohort Study
Source: JMIR Ment Health. 2026 Feb 6;13:e84318. doi: 10.2196/84318 (PMC12924039; doi:10.2196/84318)
Supplement: Multimedia Appendix 5 [file mental_v13i1e84318_app5.docx]

**Multimedia Appendix 5**

**S-Table 2**. Clinical baseline model: DASA’s Predictive Performances across different data splits (AUCs)

| **Data Timelines** | **DASA Performance (AUC)** |
| --- | --- |
| 2020-2024 | 0.6136 |
| 2020-2023 | 0.6165 |
| 2024 | 0.5934 |
